# Muscle function, Lysholm score and hop performance in individuals with clinical indications for the combined reconstruction of the anterior cruciate and the anterolateral ligaments of the knee: A cross-sectional study

**DOI:** 10.1016/j.clinsp.2023.100267

**Published:** 2023-08-17

**Authors:** Adriana Carvalho, Marilia Novaes, Juliana Sauer, Marco Kawamura Demange, Camilo Partezani Helito, Silvia Maria Amado João

**Affiliations:** a Physical Therapy Service, Instituto de Ortopedia e Traumatologia, Hospital das Clínicas, Faculdade de Medicina, Universidade de São Paulo, São Paulo, SP, Brazil; b Department of Physical Therapy, Speech and Occupational Therapy, Faculdade de Medicina, Universidade de São Paulo, São Paulo, SP, Brazil; c Knee Surgery Division, Instituto de Ortopedia e Traumatologia, Hospital das Clínicas, Faculdade de Medicina, Universidade de São Paulo, São Paulo, SP, Brazil

**Keywords:** Anterior cruciate ligament injuries, Knee injuries, Muscle strength dynamometer, Lysholm knee score

## Abstract

**Objective:**

To evaluate hip and knee muscular function, knee patient-reported outcome measures and hop performance in patients with a clinical indication for combined ACL+ALL reconstruction surgery compared to patients with an isolated ACL reconstruction surgery indication (preoperative phase) and to a control group.

**Design:**

Cross-sectional study.

**Methods:**

The sample was composed of male individuals, aged between 18 and 59 years, divided into three groups (ACL, ACL+ALL and Control). Isokinetic dynamometry was performed for the flexor and extensor knee muscles and for the hip abductors and adductors. SLHT, COHT and the Lysholm score were performed. Pain, swelling, and thigh trophism were also measured.

**Results:**

The study participants were 89 male individuals: 63 in the injury group and 26 in the control group. After applying the criteria for an ALL reconstruction indication, 33 patients were assigned to the ACL Group and 30 patients to the ACL+ALL Group. Regarding knee and hip muscle function, both groups presented worse results when compared to the control group, however, did not show significant differences compared to each other. Regarding the functional variables, the ACL+ALL group showed a significantly shorter distance achieved in the Crossover Hop Test than the other groups, as well as more pain during the tests.

**Conclusion:**

Knee and hip muscular functions are impaired after an ACL injury and do not seem to be influenced or worsened in individuals with greater rotational instability with clinical indications for combined reconstruction of the anterior cruciate and the anterolateral ligaments of the knee.

## Highlights

• Studies have shown worse rotational stability in the presence of a supposed ALL injury combined with an ACL injury. However, there is no evidence, so far, on how individuals behave regarding muscle strength and functionality.
• Knee and hip muscular functions are impaired after an ACL injury and do not seem to be influenced or worsened in individuals with greater rotational instability with clinical indications for combined reconstruction of the anterior cruciate and the anterolateral ligaments of the knee.
• The ACL+ALL group showed a significantly shorter distance achieved in the Crossover Hop Test than the other groups, as well as the reports of more pain during the tests.

## Introduction

Injury to the Anterior Cruciate Ligament (ACL) is the most common knee injury^1^ and has been an issue of major interest for researchers since the mid-19^th^ century.^2^ This injury could lead to greater rotational instability in situations such as walking and squatting when compared to healthy individuals. Kinematic, biomechanical, and imaging evaluations have shown that ACL reconstruction surgery alone may not fully restore this control, leading to residual rotational lassitude.^2^ The Anterolateral Ligament (ALL), which is one of the structures located in the anterolateral knee compartment, plays a crucial role in preventing rotational instability. First described in 1879 as a fibrous and resistant band, adjacent to the anterolateral capsule,^3^ the main function of the ALL would be to secondarily restrain internal rotation of the tibia. In ALL injury cases, there are alterations in the Pivot Shift test results, highlighted in the literature as the most reliable test to evaluate this ligament's integrity.^4^

The knee ligament injury clinical examination is crucial for diagnosis and is more effective in subacute and chronic phases, after swelling and pain reduction.^4^ Sonnery-Cottet et al.^5^ proposed some criteria to guide ACL and ALL combined reconstruction surgery based on injury history, clinical signs, and/or patient profiles. The ACL injury causes different degrees of knee dysfunction,^6^ leading to various consequences related to pain, instability, swelling and muscle strength. These alterations can affect daily living activities, although mainly recreational and sports activities.^7^

It is known that muscle strength is crucial for limb dynamic stability.^8^^,^^9^ Lower limb muscular weakness, especially in the quadriceps, is commonly related after an ACL injury,^10^^,^^11^ representing one of the major losses for these individuals.^12^^,^^13^ The functional deficit in the quadriceps is three times greater in relation to the hamstrings.^14^ Studies have shown worse rotational stability in the presence of a supposed ALL injury combined with an ACL injury.^15^^,^^16^ However, there is no evidence, so far, on how individuals behave regarding muscle strength and functionality.

Due to an increase in indications for combined ACL and ALL reconstruction surgery,^17^ it has become necessary to understand the possible deficits and muscular function alterations in candidates for this procedure. While alterations following an isolated ACL injury are well understood, there is still limited understanding of these alterations in candidates for the combined reconstruction.

Based on this context, some questions were raised regarding individuals with clinical indications for combined ACL and ALL reconstruction, mainly in relation to muscle function and functionality. The hypotheses were that individuals with a combined ACL+ALL reconstruction surgery indication would present greater muscle function deficits, as well as lower Lysholm scores and hop test performances when compared to individuals with an isolated ACL reconstruction surgery indication.

In summary, the goal of the present study was to evaluate hip and knee muscular function, knee patient-reported outcome measures and hop performance in patients with a clinical indication for combined ACL+ALL reconstruction surgery compared to an isolated ACL reconstruction surgery indication and to a control group. In addition, the aim was to evaluate knee pain, swelling and thigh trophism and to verify the correlation of knee muscle function with time since injury, Lysholm score, Single Leg Hop Test, Crossover Hop Test and pain.

## Methods

### Study design

This was a cross-sectional study approved by the ethics committee of the study institution (nº 54541116.8.0000.0068), with informed consent obtained from each participant. The evaluations were performed between January 2018 and December 2019.

The sample size was based on a pilot study and the following assumptions were used: a 5% type I error, a study power of 0.8 and a statistical difference of at least 15% between groups to indicate clinical significance. The primary outcome used for the calculation was knee extensor peak torque corrected for body weight at 60°/s. Data obtained from the control group in the pilot study were used as a reference, with a mean of 260 Nm and a standard deviation of 70 Nm. A loss of up to 10% of sample data was also considered. Accordingly, it was necessary to have a sample size of at least 30 subjects in each group.

### Participants

The sample was composed of male individuals, aged between 18 and 59 years, divided into three groups (Control Group; ACL Group and ACL+ALL Group). Patients on the institute's waiting list for ACL reconstruction were invited to participate in the study. Individuals in the Control Group were volunteers with no formal link to the institution.

The following inclusion criteria were adopted:

• ACL Group: ACL injury confirmed by clinical examination and by a Magnetic Resonance Imaging (MRI) exam, evaluated by a musculoskeletal radiologist and a knee surgeon.
• ACL+ALL Group: patients with an ACL injury (as described above) and also a pivot-shift test grade III and/or the presence of two of the following criteria: less than 20 years of age, time since injury over 1 year, anterior tibial translation difference between legs greater than 7 mm.^5^^,^^18^
• Control Group: healthy individuals, with no previous history of musculoskeletal disease.

None of the individuals were professional athletes. Except for the ACL+ALL Group, all participants were free from multi-ligament injuries, advanced tibiofemoral and patellofemoral osteoarthritis (with an articular axle deviation), or other musculoskeletal diseases.^19^ Patients with a meniscal injury were not excluded from the study, due to the fact that this injury is frequently associated with an ACL injury.

None of the participants had received physical therapy treatment after the injury and the International Physical Activity Questionnaire (short version) was used to characterize the subjects’ physical activity level. Regular physical activity was defined as participants performing an average of between 150 and 300 min of moderate-intensity, or between 75 and 150 min of vigorous-intensity physical activity per week.^20^

### Procedures

#### Anterior tibial translation

The anterior tibial translation was measured using the KT-1000 knee arthrometer^TM^ (MEDmetric, San Diego, CA). The subject was placed in the supine position with the cushion (provided with the equipment) placed under the thighs to maintain the knees flexed at approximately 30°. Before each test, the device was recalibrated to zero, and the anterior tibial translation measurement was performed. The equipment calculated the tibial displacement in mm. The arithmetic mean of the results of three tests was calculated for each leg.^21^

#### Muscular function

**Fig. 1:**
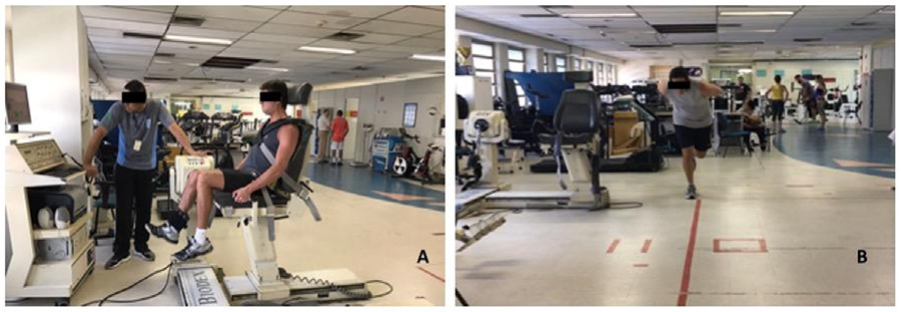
Muscle function evaluation (A) and hop test (B).

Isokinetic dynamometry was performed utilizing the Biodex® Multi-Joint System 3 (Biodex Medical, Shirley, NY, USA) (Fig. 1A) to register the Peak Torque corrected for Body Weight (PT/BW%), Total Work (TW), and agonist/antagonist relation (%) for the flexor and extensor knee muscles at angular velocity of 60°/s and 120°/s; PT/BW% and TW for hip abductors and adductors at 60°/s. The evaluation was carried out in five steps, according to Greve.^22^

#### Knee patient-reported outcome measure and hop performance

The Lysholm scale was used to evaluate knee function, considering eight different domains related to the knee: limping, support, restraining, instability, pain, swelling, climbing stairs, and squatting. Each domain has closed answer alternatives, and the final score is expressed nominally and ordinally, with a score ranging from 95 to 100 points regarded as “excellent”, 84 to 94 points, “good”, from 65 to 83 points, “fair”, and “poor” when values are 64 points or less.^23^

For the hop tests, a 6-meter long and 15-cm wide line on the floor was used. The anterior extremity of the foot was placed on the line starting point. For both injury groups, the participants were instructed to initiate the tests with the contralateral limb. Healthy subjects started with the dominant leg. Before each test, the participants performed two trials to become familiar with the movement, followed by three official trials with the results recorded for both limbs. All participants were instructed to keep their arms crossed behind their backs while performing the tasks (Fig. 1B).

The Single Leg Hop Test (SLHT) consisted of one leg hop, trying to jump as far as possible along the line, landing with only one foot on the floor. For the Crossover Hop Test (COHT), participants performed three consecutive hops crossing the line.^24^^,^^25^ The distance between the heel and the line starting point was evaluated with a measuring tape for each trial. The arithmetic mean of three trials was utilized for statistical purposes.

#### Pain, swelling and thigh trophism

During the tests, pain was measured using the 10-point Numerical Rating Scale (NRS). The participants also answered “yes” or “no” when asked about any sign of instability when landing. In addition, the NRS was used to evaluate the knee at rest prior to the tests. Patients were instructed to classify their pain from 0 (meaning no pain) to 10 (maximum possible pain).^26^

Knee circumference was measured with a measuring tape at the joint interline to evaluate the swelling and 10 cm above the patella to examine the thigh muscle trophism,^27^ with the participant in the supine position, with the thigh relaxed.

#### Data analysis

Data were stored in an Excel data sheet (version for Mac) and imported into the SPSS® 25 for MAC Software to carry out the statistical analysis. Categorical data were described by their absolute and relative frequencies and continuous data were tested for their distribution through the Shapiro-Wilk test and by subjective histogram graphical analysis. Each group was described separately, and the Chi-Squared test was used for the comparison of the categorical data between groups. The continuous data showed asymmetrical distribution. Therefore, the Kruskal-Wallis was chosen for the comparison between the three groups. For the post-hoc pair comparison, the Mann-Whitney test with Bonferroni correction was adopted.

For the data with normal distribution, the Spearman's correlation test was utilized to evaluate the relation between knee muscular function and the following variables: time since injury, pain, Lysholm score, Single Leg Hop Test, and Crossover Hop Test; defined by the following index: very low (0‒0.29); low (0.30‒0.49); moderate (0.50‒0.69); strong (0.70‒0.89) and very strong (0.90‒1.00).^28^

Aiming to evaluate the statistical significance, a type I error ≤ 0.05 was adopted.

## Results

The study participants were 89 male individuals: 63 in the injury group and 26 in the control group. The number of eligible, included, and excluded patients is detailed in the flowchart (Appendix).

After the group's division ‒ by applying the previously stated criteria ‒ the ACL Group included a total of 33 patients and the ACL+ALL Group 30 patients.

Group demographics are reported in Table 1. There was no significant difference between the groups regarding age, weight, physical activity level, and dominance; except for height, where the participants of the ACL and Control groups were, on average, taller than those of the ACL+ALL Group.

**Table 1:** Demographic data of enrolled patients.

	ACL Group (*n* = 33)	ACL+ALL Group (*n* = 30)	Control Group (*n* = 26)	*p*-value
**Age (years)**	27 ± 8	30 ± 10	28 ± 3	0.506
**Height (cm)**	177 ± 7^a^	174 ± 7	178 ± 8^a^	0.019
**Weight (kg)**	82 ± 13	79 ± 13	82 ± 13	0.553
**Physically active individuals (n)**	25 (83%)	20 (61%)	21 (81%)	0.079
**Dominance (right dominance %)**	27 (90%)	28 (85%)	23 (88%)	0.815
**Time since injury (months)**	33 ± 36	31 ± 37	‒	<0.001
**Lateral meniscus injury (n)**	3 (10%)	9 (27%)	0 (0%)	0.165
**Medial meniscus injury (n)**	13 (43%)	9 (27.3%)	0 (0%)	0.165
**Right limb injured (%)**	19 (63%)	14 (42%)		0.147
**Associated injuries (%)**	20 (67%)	21 (64%)	‒	0.742
**Injury during sports activity (% of individuals)**	27 (90%)	29 (88%)	2 (100%)	0.653

a *p* < 0.05 compared to the ACL+ALL Group. † *p* < 0.05 compared to the Control Group. ¥ *p* < 0.05 compared to the ACL Group. ACL+ALL, Combined Reconstruction Indication Group; LCA, Isolated Reconstruction Indication Group; cm, centimeters; kg, kilograms.

The ACL Group presented more time since the injury compared to the ACL+ALL Group (*p* < 0.001). Regarding pain, knee and thigh circumference, there was no statistically significant difference between the groups. The ACL+ALL Group patients achieved shorter distances in the COHT than the ACL Group and the Control Group and also presented a greater number of individuals with pain complaints during both hop tests (Table 2). Regarding muscular function, there were no statistically significant differences for the affected limb (Tables 3 and 4).

**Table 2.** Knee patient-reported outcome measure and hop performance.

	ACL Group	ACL+ALL Group	Control Group	*p*-value
Lysholm score	72 ± 15^b^	68 ± 21^b^	98 ± 4	<0.001
SLHT distance (cm) AL	101 ± 33^b^	83 ± 38^b^	129 ± 25	<0.001
SLHT distance (cm) CL	129 ± 28	119 ± 49^b^	130 ± 23	0.041
COHT distance (cm) AL	285 ± 92^a^^,^^b^	215 ± 104^b^	400 ± 92	<0.001
COHT distance (cm) CL	325 ± 89^b^	285 ± 96^b^	400 ± 92	<0.001
SLHT pain AL (%)	6 (21%)^a^^,^^b^	16 (48%)^b^	2 (4%)	<0.001
SLHT pain CL	0 (0%)	0 (0%)	2 (4%)	0.295
COHT pain AL (%)	7 (25%)^a^^,^^b^	16 (50%)^b^	1 (2%)	<0.001
COHT pain CL	0 (0%)	0 (0%)	1 (2%)	0.552
SLHT instability AL (%)	10(36%)^b^	15(45%)^b^	1(2%)	<0.001
SLHT instability CL (%)	0 (0%)	0 (0%)	0 (0%)	NA
COHT instability AL (%)	10 (36%)^b^	17 (53%)^b^	1 (2%)	<0.001
COHT instability CL	1 (4%)	1 (3%)	0 (0%)	0.417

a *p <* 0.05 compared to the ACL+ALL Group.

b *p <* 0.05 compared to the Control Group. ¥ *p <* 0.05 compared to the ACL Group. ACL+ALL, Combined Reconstruction Indication Group; LCA, Isolated Reconstruction Indication Group; NRS, Numerical Rating Scale; cm, centimeters; kg, kilograms; AL, Affected Limb; CL, Contralateral Limb; SLHT, Simple Leg Hop Test; COHT, Crossover Hop Test; NA, Not Applicable.

**Table 3.** Muscular function evaluation for affected limb ‒ 60°/s.

Iso 60°/s	ACL Group	ACL+ALL Group	Control Group	p-value
Hip Abductors PT/BW (%)	135 ± 44	131 ± 42	129 ± 39	0.821
Hip Abductors TW (J)	284 ± 81	242 ± 79	272 ± 60	0.081
Knee Extensors PT/BW (%)	195 ± 49^b^	220 ± 69^b^	260 ± 71	0.000
Knee Flexors PT/BW (%)	128 ± 69	118 ± 50	127 ± 36	0.246
Knee Extensors TW (J)	657 ± 201^b^	676 ± 211^b^	886 ± 216	0.000
Knee Flexors TW (J)	385 ± 122	335 ± 148	472 ± 113	0.691
Knee Ag/Ant (%)	58 ± 13^a^^,^^b^	49 ± 13	49 ± 7	0.016

a *p <* 0.05 compared to the ACL+ALL Group.

b *p <* 0.05 compared to the Control Group. ^¥^*p <* 0.05 compared to the ACL Group. ACL+ALL, Combined Reconstruction Indication Group; LCA, Isolated Reconstruction Indication Group; Iso, Isokinetic; PT/BW, Peak Torque/Body Weight; TW, Total Work; J, Joules; Ag/Ant, Agonist/Antagonist relation.

**Table 4.** Muscular function evaluation on affected limb ‒ 120°/s.

Iso 120°/s	ACL Group	ACL+ ALL Group	Control Group	p-value
Knee Extensors PT/BW (%)	176 ± 38^†^	192 ± 47	221 ± 45	0.003
Knee Flexors PT/BW (%)	97 ± 21^†^	100 ± 33	117 ± 28	0.032
Knee Extensors TW (J)	575.69 ± 163.31^†^	586 ± 157^†^	749 ± 168	0.000
Knee Flexors TW (J)	329 ± 99	303 ± 113^†^	400 ± 102	0.008
Knee Ag/Ant (%)	56 ± 11	52 ± 12	53 ± 7	0.411

* *p <* 0.05 compared to the LCA+LAL Group.

* *p <* 0.05 compared to the ACL+ALL Group.

† *p <* 0.05 compared to the Control Group.

¥ *p <* 0.05 compared to the ACL Group.

ACL+ALL, Combined Reconstruction Indication Group; LCA, Isolated Reconstruction Indication Group; Iso, Isokinetic; PT/BW, Peak Torque/Body Weight; TW, Total Work; J, Joules; Ag/Ant, Agonist/Antagonist relation.

Correlation data are shown in Table 5, there was a strong correlation between knee extensor peak torque corrected for body weight at 120°/s and the SLHT (0.7) (Table 5).

**Table 5.** Correlation analysis.

Isokinetic evaluation	Time since injury (months)	Lysholm score	SLHT (cm)	COHT (cm)	NRS (0‒10)
Knee Extensors	−0.017	0.414	0.579^a^	0.528^a^	−0.094
PT/BW (%)					
60°/s					
Affected limb					
Knee Flexors	−0.059	0.210	0.480	0.421	−0.111
PT/BW (%)					
60°/s					
Affected limb					
Knee Extensors	0.185	0.425	0.464	0.476	0.027
TW (J)					
60º/s					
Affected limb					
Knee Flexors	0.055	0.407	0.447	0.535^a^	−0.037
TW (J)					
60°/s					
Affected limb					
Ag/Ant	−0.232	−0.101	−0.022	0.036	−0.051
60°/s					
Affected limb					
Knee Extensors	−0.045	0.309	0.700^b^	0.632^a^	−0.105
PT/BW (%)					
120°/s					
Affected limb					
Knee Flexors	−0.092	0.233	0.566^a^	0.579^a^	0.011
PT/BW (%)					
120°/s					
Affected limb					
Knee Extensors	0.087	0.301	0.529^a^	0.544^a^	0.081
TW (J)					
120°/s					
Affected limb					
Knee Flexors	0.005	0.200	0.396	0.503^a^	0.164
TW (J)					
120°/s					
Affected limb					
Ag/Ant	−0.070	−0.003	−0.091	0.022	0.196
60°/s					
Affected limb					

a Moderate correlation coefficient.

b Strong correlation coefficient. PT/BW, Peak Torque/Body Weight; TW, Total Work; J, Joules; Ag/Ant, Agonist/Antagonist relation; cm, Distance measured in centimeters; SLHT, Single Leg Hop Test; COHT, Crossover Hop Test; NRS, Numerical Rating Scale.

## Discussion

The aim of the study was to evaluate hip and knee muscular function, knee patient-reported outcome measures, and hop performance in patients with a clinical indication for combined ACL+ALL reconstruction surgery compared to an isolated ACL reconstruction surgery indication and to a control group. In addition, the study aimed to evaluate knee pain, swelling and thigh trophism.

The groups showed some differences regarding their performance in the hop tests: in the Crossover Hop Test, the ACL+ALL Group participants achieved a significantly shorter distance than the ACL and Control Groups. This result could be explained by the fact that this type of activity requires greater rotational stability control. This control may have been altered in the ACL+ALL Group, leading to an increase in tibial internal rotation^9^ and, therefore, to greater dynamic knee valgus.

A plausible explanation could be the presence of a greater neuromuscular control deficit in the patients of the ACL+ALL group. This control is already altered after an isolated ACL injury, as are movement patterns and the ability to stabilize the knee during dynamic activity.^29^^,^^30^ Although this difference is known to exist in patients with ACL injuries, the deficits were even greater in the ACL+ALL Group. Furthermore, these changes were considerably more noticeable during the Crossover Hop Test, a dynamic activity that requires greater rotational stability. This result was not found in linear activities, such as the Single Hop Test.

Another possible reason for this finding could be the presence of pain during the hop tests. Although both injury groups reported pain, the ACL+ALL Group showed a higher proportion of this symptom in both the Single and Crossover Hop Tests.

After an ACL injury, Kapreli et al.,^31^ found central nervous system dysfunction, verified by MRI, in the SII cortical area responsible for sensory stimulus reception, which could explain the presence of pain during these movements. Therefore, the presence of pain could have interfered with the hop test performances, considering that it can also affect functionality, as previously described by Negahban et al.^32^ Furthermore, the work of Pua et al.^33^ showed that Single Leg Hop Test performance is independent of conventional muscular knee function evaluation, age, gender, and knee pain, which could explain why the Crossover Hop Test was the only altered evaluation in the present study.

Considering muscular function, it was hypothesized that greater knee rotational instability would lead to an increase in knee and hip deficits. However, the results did not confirm this hypothesis. Both injury groups presented worse results when compared to the control group; however, they did not show significant differences between each other. A possible explanation for this finding could be the fact that an isolated ACL injury already leads to devastating effects on knee muscular function, especially on the extensor mechanism, supported by previous studies.^14^^,^^34^, ^35^, ^36^ After the injury, arthrogenic muscle inhibition occurs as a natural compensation mechanism to avoid excessive anterior tibial displacement, leading to pain and movement restriction.^13^^,^^37^^,^^38^ However, it is important to highlight that such evaluations are linear, executed in only one anatomical plane.

Regarding the correlations between knee extensor peak torque 120°/s and the SLHT, it can be deduced that the stronger the knee extensor muscles, the greater the hop performances will be. Both injury groups achieved a shorter distance when compared to the control group, which could be explained by the quadriceps strength deficit ‒ which equates to a lower capacity for absorbing and generating power during the activity.^39^ The present results are in line with those found in the literature.^13^^,^^35^^,^^40^ The study by Keays et al.^35^ also reinforces these findings by showing a positive correlation between muscular function and functional stability.

## Conclusion

No differences were found between the ACL+ALL and the ACL groups regarding knee and hip muscular functions or for the functional score, pain, swelling, and thigh trophism variables. The ACL+ALL Group showed a significantly shorter distance achieved in the Crossover Hop Test than the other groups, as well as the reports of more pain during the tests. There was a strong correlation between knee extensor peak torque corrected for body weight at 120°/s and the SLHT.

### Study limitations

The inclusion of only men allowed the sample to be homogeneous, and to avoid gender differences affecting the results. However, this constitutes a study limitation because gender-related risk factors show female populations to have a higher predisposition to ACL injury than males.^41^ The results are restricted to the studied population; more studies are needed with women to understand how they behave within the criteria studied here.

## Conflicts of interest

The authors declare no conflicts of interest.

## Appendix Supplementary materials

Image, application 1
