# Supplementary material for: Muscle function, Lysholm score and hop performance in individuals with clinical indications for the combined reconstruction of the anterior cruciate and the anterolateral ligaments of the knee: A cross-sectional study
Source: Clinics (Sao Paulo). 2023 Aug 17;78:100267. doi: 10.1016/j.clinsp.2023.100267 (PMC10460945; doi:10.1016/j.clinsp.2023.100267)
Supplement: Supplementary file 1 [file mmc1.docx]

**CLINICS-D-22-00660_Supplementary Material**

**Appendix**
